# Impact of IL6 and IL18 Promoter Polymorphisms on Circulating Cytokine Levels, Gene Expression, and Susceptibility to Rheumatoid Arthritis

**DOI:** 10.3390/ijms27167132

**Published:** 2026-08-09

**Authors:** Viktoria Vasileva, Georgi Vasilev, Mariana Ivanova, Lyuba Miteva, Irena Manolova

**Affiliations:** 1 Department of Molecular Biology, Immunology and Medical Genetics, Medical Faculty, Trakia University, 6000 Stara Zagora, Bulgaria; dr.viktoriya.vasileva@trakia-uni.bg (V.V.); lyuba.miteva@trakia-uni.bg (L.M.); 2 Clinical Laboratory, University Hospital Trakia, 6000 Stara Zagora, Bulgaria; 3 Laboratory of Immunology, Specialized Hospital for Active Treatment of Hematological Diseases, 1A, Kliment Ohridski Blvd., 1797 Sofia, Bulgaria; drgeorgivasilev@gmail.com; 4 Clinic of Rheumatology, University Hospital “St. Ivan Rilski”, 1612 Sofia, Bulgaria; mariana_ig@abv.bg; 5 Medical Faculty, Medical University, 1413 Sofia, Bulgaria

**Keywords:** gene expression, rheumatoid arthritis, IL-6, IL-18, rs1800795, rs1946518

## Abstract

Our study examined the impact of *IL6* −174 G/C (rs1800795) and *IL18* −607 C/A (rs1946518) promoter polymorphisms on susceptibility to rheumatoid arthritis (RA) and protein/mRNA levels. RA patients had significantly higher serum IL-6 and IL-18 levels than controls; healthy men had higher IL-18 than women (*p* < 0.001). Conversely, *IL6* mRNA expression was lower in RA patients than controls (*p* = 0.037). No link was found between rs1800795 and RA susceptibility; however, the CC genotype was less frequent among RA patients than controls, suggesting potential protection (OR = 0.55). Also, the −174 G allele was probably associated with RF positivity (OR = 1.4). In RA patients, no significant association was detected between the −174 G/C polymorphism and either *IL6* mRNA expression or circulating IL-6 levels. Among healthy individuals, carriers of the GG genotype showed higher *IL6* mRNA expression (*p* = 0.024). No association was observed between rs1946518 and RA risk or clinical features, or between systemic *IL18* mRNA, IL-18 levels, and rs1946518 genotypes in RA patients. In summary, rs1800795 showed borderline association with disease risk and autoantibody presence, whereas rs1946518 was not associated with RA susceptibility, cytokine levels, or key clinical characteristics in our cohort.

## 1. Introduction

Rheumatoid arthritis (RA) is a systemic immune-mediated disorder characterised by persistent synovial inflammation and progressive joint damage. While a definitive diagnosis necessitates clinical arthritis, many individuals with RA experience joint-related complaints before the appearance of swelling and unmistakable signs of arthritis [1]. In the affected joints, cells such as neutrophils, T and B lymphocytes, macrophages, osteoclasts, and synovial fibroblasts become extensively activated, triggering the release of a wide range of cytokines and chemokines [2]. These pro-inflammatory mediators stimulate the production of enzymes and other effector molecules that drive progressive cartilage and bone damage. Simultaneously, the heightened immune response extends beyond the joints, causing inflammatory changes in multiple organs—including the heart, lungs, and blood vessels—often resulting in long-term disability and an increased risk of premature death [3]. The importance of these pathogenic cytokines becomes clearer when considering newer ideas that aim to group diseases based on how pro-inflammatory cytokine pathways are organized and interact. Considering this approach, it highlights that several autoimmune conditions show similar disruptions in particular signaling pathways, which may represent specific vulnerabilities that underpin the inflammatory process [4].

In our previous study analyzing serum cytokine profiles in RA patients, we reported significantly elevated levels of pro-inflammatory cytokines interleukin-6 (IL-6), IL-12p40, IL-17A, IL-18, IL-23, and Tumor Necrosis Factor-alpha (TNF-α), with IL-6 and IL-18 being particularly important, along with T-helper17 (Th17) axis cytokines [5]. IL-6 and IL-18 are among the main pathogenic cytokines in RA, contributing significantly to both local tissue damage and systemic symptoms. IL-18 is a potent pro-inflammatory cytokine that is part of the IL-1 superfamily [6]. It is secreted by both hematopoietic and non-hematopoietic cells, such as monocytes, macrophages, osteoblasts, keratinocytes, mesenchymal cells, and dendritic cells [7]. We identified IL-18 as a key factor in the pathogenesis of RA, especially in women. We emphasized its role as one of the most consistently and significantly dysregulated cytokines, positioning it as a “vulnerable node” within the cytokine network of this systemic autoimmune disease [5]. IL-6, a multifunctional cytokine and member of the IL-6 cytokine family, is synthesized by various cell types, including monocytes, macrophages, fibroblasts, endothelial cells, synovial cells, and chondrocytes [8]. It is involved in several biological processes, including B-cell differentiation, T-cell activation, the induction of acute-phase proteins, and osteoclastogenesis [9]. IL-6 is recognized as a pivotal cytokine that significantly contributes to joint and systemic inflammation in RA [10], consistent with our data showing an association between IL-6 levels and higher disease activity [5].

In this context, we concentrated on the roles of IL-6 and IL-18 in RA, particularly regarding how genetic background impacts their levels, which is essential for RA development and clinical manifestations. The expression of IL-6 and IL-18 is influenced by polymorphisms, predominantly within their promoter regions, which are typically highly polymorphic and play a role in regulating transcription and translation. The IL-6 gene is located on chromosome 7p15.3, and numerous promoter polymorphisms have been studied for their potential impact on IL-6 transcription and circulating cytokine levels. One of the most frequently investigated variants is *IL6* −174 G/C (rs1800795), which has been linked to altered gene expression and has been widely studied for its potential role in influencing susceptibility to RA and its clinicopathological characteristics in European and Asian populations. However, the available evidence remains inconsistent [3,11]. Similarly, the −607 C/A variant in the *IL18* promoter has been implicated in RA risk [12,13]. The gene encoding IL-18 is located on chromosome 11q23.1 and the most frequently searched SNP of the IL-18 gene is *IL18* −607 C/A (rs1946518). However, there is conflicting data on the impact of this polymorphism on the incidence and severity of RA [3].

The present study aims to examine the association of the *IL6* −174 G/C (rs1800795) and *IL18* -607 C/A (rs1946518) genetic variants with susceptibility to RA, along with the clinical characteristics and their potential impact on gene expression at the protein and mRNA levels in the Bulgarian population.

## 2. Results

### 2.1. Characteristics of Study Subjects

The RA group comprised 159 patients (11 males and 148 females) with a mean (±SD) age of 55.4 ± 11.8 years, compared to 188 healthy controls (66 males and 122 females) with a mean (±SD) age of 52.8 ± 12.4 years. The mean (±SD) disease duration was 8.9 ± 8.6 years. Seventy-three per cent of RA patients tested positive for rheumatoid factor (RF-IgM), and 61.0% tested positive for anti-cyclic citrullinated peptide antibody (anti-CCP). Disease activity was classified as follows: low disease activity/remission (DAS28-CRP < 3.2) in 18 patients (11.3%); moderate activity (DAS28-CRP between 3.2 and 5.1) in 94 patients (59.1%); and high activity (DAS28-CRP > 5.1) in 23 patients (29.6%). Regarding drug therapy, 53 patients (33.3%) received only symptomatic treatments, including long-term low-dose prednisolone at 10 mg/day. In comparison, 83 patients (52.2%) were treated with conventional synthetic disease-modifying anti-rheumatic drugs (csDMARDs), and 23 patients (14.5%) received biologic disease-modifying anti-rheumatic drugs (bDMARDs). All participants were of white Caucasian ancestry. The demographic and clinical characteristics of RA patients are summarized in Table 1.

### 2.2. Association of IL18 −607 C/A (rs1946518) and IL6 −174 C/G (rs1800795) SNPs with Susceptibility to RA

Genotype and allele frequencies of rs1946518 and rs1800795 among RA patients and healthy controls are presented in Table 2. The genotype distribution of *IL6* −174 C/G and *IL18* −607 C/A was in agreement with Hardy–Weinberg equilibrium (HWE) among RA cases and controls, with all *p*-values greater than 0.05.

Analysis of the IL18 −607 C/A polymorphism in our study cohort revealed no statistically significant differences between cases and controls concerning genotype distribution (*p* = 0.72) and allele frequencies (*p* = 0.26). No association between the rs1946518 polymorphism and RA risk was established under the co-dominant model (AA vs. CC; OR = 1.23, 95% CI = 0.55–2.73), dominant model (CA + AA vs. CC; OR = 1.21, 95% CI = 0.76–1.91), recessive model (AA vs. CC + CA; OR = 1.11, 95% CI = 0.52–2.36), over-dominant model (CA vs. CC + AA; OR = 1.16, 95% CI = 0.74–1.81), and log-additive model (OR = 1.14, 95% CI = 0.80–1.63). No association between the IL6 rs1800795 polymorphism and RA risk was established under the allelic model (OR = 0.82, 95% CI = 0.62–1.09), the co-dominant model (CC vs. GG; OR = 0.55, 95% CI = 0.26–1.15), the dominant model (CG + CC vs. GG; OR = 0.88, 95% CI = 0.56–1.39), the over-dominant model (CG vs. CC + GG; OR = 1.14, 95% CI = 0.73–1.8) and the log-additive model (OR = 0.81, 95% CI = 0.58–13). Nevertheless, we found the homozygous CC genotype was less frequent in RA cases (9.4%) compared to healthy controls (15.9%) and a tendency for genetic predisposition under the recessive model (CC vs. GG + CG; OR = 0.55, 95% CI = 0.27–1.10, *p* = 0.087). Our findings suggest that the GG + CG genotypes may confer a greater risk of RA in the Bulgarian population than the CC genotype. Statistical power analysis was performed. A sensitivity analysis using G*Power 3.1 (α = 0.05, total sample size = 347, df = 2) indicated that the present study had 80% statistical power to detect a minimum effect size of Cohen’s w = XX in the primary genetic association analyses. The observed effect sizes were Cohen’s w = 0.073 for *IL18* rs1946518 and Cohen’s w = 0.091 for *IL6* rs1800795.

To evaluate the potential interaction between genetic variants at these loci and RA susceptibility, a combined two-locus model was constructed using dichotomous variables for each cytokine gene polymorphism (Supplementary Table S1). For *IL18* −607 C/A, we used the dominant model, and for *IL6* −174 C/G, we used the recessive model, thereby allowing for assessment of four combined genotypes. No statistically significant association was observed between the combined genotypes of *IL18* −607 C/A and *IL6* −174 C/G SNPs and RA (χ^2^ = 6.018; *p* = 0.111; 4 × 2 contingency table), irrespective of homo- and heterozygous genotypes.

### 2.3. Associations Between IL18 −607 C/A (rs1946518) and IL6 −174 C/G (rs1800795) SNPs and RA Disease Characteristics

The disease characteristics of RA patients were analysed in relation to the genotypes of *IL18* −607 C/A (rs1946518) and *IL6* −174 C/G (rs1800795) SNPs. RA patients carrying the *IL6* −174 G allele (CG + GG) were more likely to be RF-positive than those with the homozygous CC genotype (75.0% vs. 53.3%, OR = 1.4, 95% CI = 0.86–2.28, *p* = 0.072). No association of rs1800795 with anti-CCP positivity was found, nor was there an association of rs1946518 with autoantibody status. There were no significant links between rs1946518 and rs1800795 and age, disease onset, disease duration, DAS28-CRP, or CRP levels. Data are presented in Table 3.

### 2.4. Serum Levels of IL-18 and IL-6 in Relation to IL18 −607 C/A (rs1946518) and IL6 −174 C/G (rs1800795) Gene Polymorphisms

Serum levels of both cytokines were measured in 89 RA patients (9 males and 80 females) and 89 healthy controls (39 males and 50 females). The concentrations are shown in Table 4. The serum cytokine levels in RA patients exhibited a right-skewed distribution, as expected considering the heterogeneity of the RA cohort analysed. The natural logarithm of circulating cytokine levels showed a Gaussian distribution and was used in the linear regression analysis.

IL-18 serum levels were significantly higher in RA patients, with an average of 222.9 ± 265.6 pg/mL (mean ± SD), compared to healthy controls, who had levels of 128.9 ± 76.7 pg/mL (mean ± SD) (*p* < 0.001). Analysis of the study population by gender revealed that IL-18 serum levels were significantly higher in healthy men compared to healthy women (161.7 ± 75.4 pg/mL vs. 96.7 ± 63.5 pg/mL, *p* < 0.001), but no significant difference was found between males and females within the patient group (220.3 ± 143.4 pg/mL vs. 223.2 ± 273.5 pg/mL, *p* = 0.573). Accordingly, no significant difference in IL-18 levels was found between male RA patients and healthy men (220.3 ± 143.4 pg/mL vs. 161.7 ± 75.4 pg/mL, *p* = 0.204), whereas female RA patients showed significantly higher IL-18 levels than healthy women (223.2 ± 273.5 pg/mL vs. 96.7 ± 63.5 pg/mL, *p* < 0.001) (Table 4).

Data presented in Table 5 show the IL-18 serum concentrations in relation to the *IL18* gene polymorphisms. No significant differences in cytokine levels stratified by genotypes of the studied polymorphisms were found in both RA patients (*p* = 0.302) and the control group (*p* = 0.183, Kruskal–Wallis test for K independent samples). In the control group, the −607 CA genotype was linked to higher IL-18 production (143.4 ± 78.1 pg/mL) compared to the −607 AA genotype (88.0 ± 56.4 pg/mL) (*p* = 0.078, Mann–Whitney U test), but this difference disappeared when taking into account only healthy women (Table 6). Inter-group comparisons showed that IL-18 serum levels were elevated in RA patients carrying the −607 CC and −607 AA genotypes compared with control subjects with the same genotypes (*p* = 0.014 and *p* = 0.009, respectively). Stratified analysis revealed similar findings in women with the −607 CC (*p* = 0.002), −607 CA (*p* = 0.002), and −607 AA genotypes (*p* = 0.02) (Table 6). The results from the multivariate general linear models using the natural logarithm of IL-18 levels indicated that genotype and age do not influence cytokine level in the healthy subject group, whereas male gender was associated with higher levels of IL-18 (β = 0.487; *p* < 0.001) (Supplementary Table S2). In the RA patient group the genotype, age, and gender showed no effect on log-transformed IL-18 levels. The results were consistent with the results obtained using the Mann–Whitney U test of non-transformed IL-18 values.

When comparing serum IL-6 levels between RA patients and healthy controls (Table 4), the patient group showed significantly higher cytokine levels, with an average of 23.6 ± 38.9 pg/mL (mean ± SD), whereas controls had an average of 1.8 ± 2.4 pg/mL (mean ± SD) (*p* < 0.001). Sex-based analyses revealed significant differences between patients and controls in both females (*p* < 0.001) and males (*p* = 0.004). Regarding IL-6 levels in relation to rs1800795 genotypes, no significant differences between the three genotypes were found in either cases (*p* = 0.572) or controls (*p* = 0.148), as shown in Table 5 (Kruskal–Wallis test for K independent samples). Genotype-stratified analysis revealed that serum IL-6 levels were significantly higher in patients compared to controls with the same genotype (Table 5).

Healthy controls carrying the −174 CC genotype showed lower IL-6 production than those with the −174 GG (*p* = 0.116, Mann–Whitney U test) and −174 CG (*p* = 0.056, Mann–Whitney U test) genotypes, but these differences did not reach statistical significance. The results from the general linear models showed that genotype, age, and gender had no effect on the natural log of IL-6 levels in healthy subjects and RA patients (*p* > 0.05) (Supplementary Table S3). These results were in compliance with the Mann–Whitney U test of non-transformed IL-6 levels.

### 2.5. Gene Expression of IL18 and IL6 mRNA in Peripheral Blood

**Figure 1:**
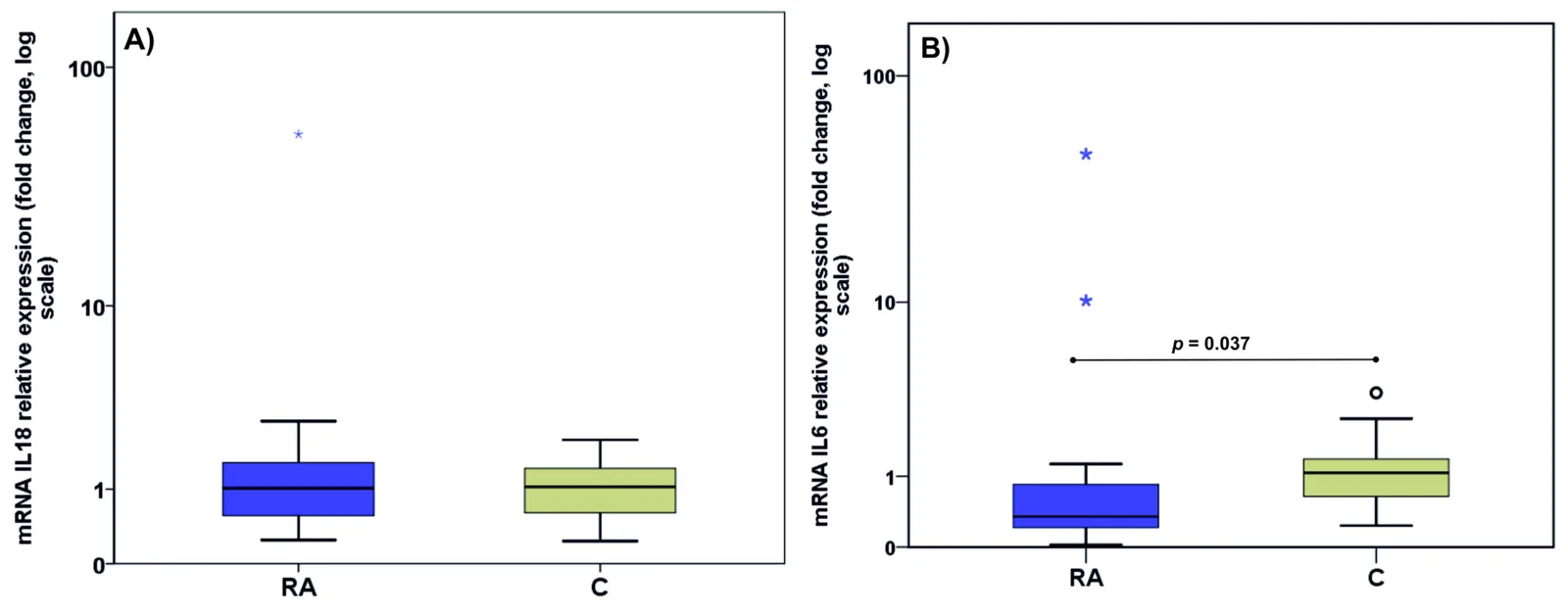
Relative mRNA expression levels of *IL18* (**A**) and *IL6* (**B**) in female patients with rheumatoid arthritis (RA) and healthy controls (C). Data are presented as the median with the lower (Q1) and upper (Q3) quartiles. Outliers are indicated by circles, and extreme values are indicated by asterisks. The *y*-axis is displayed on a logarithmic scale. extreme values are indicated by asterisks.

**Figure 2:**
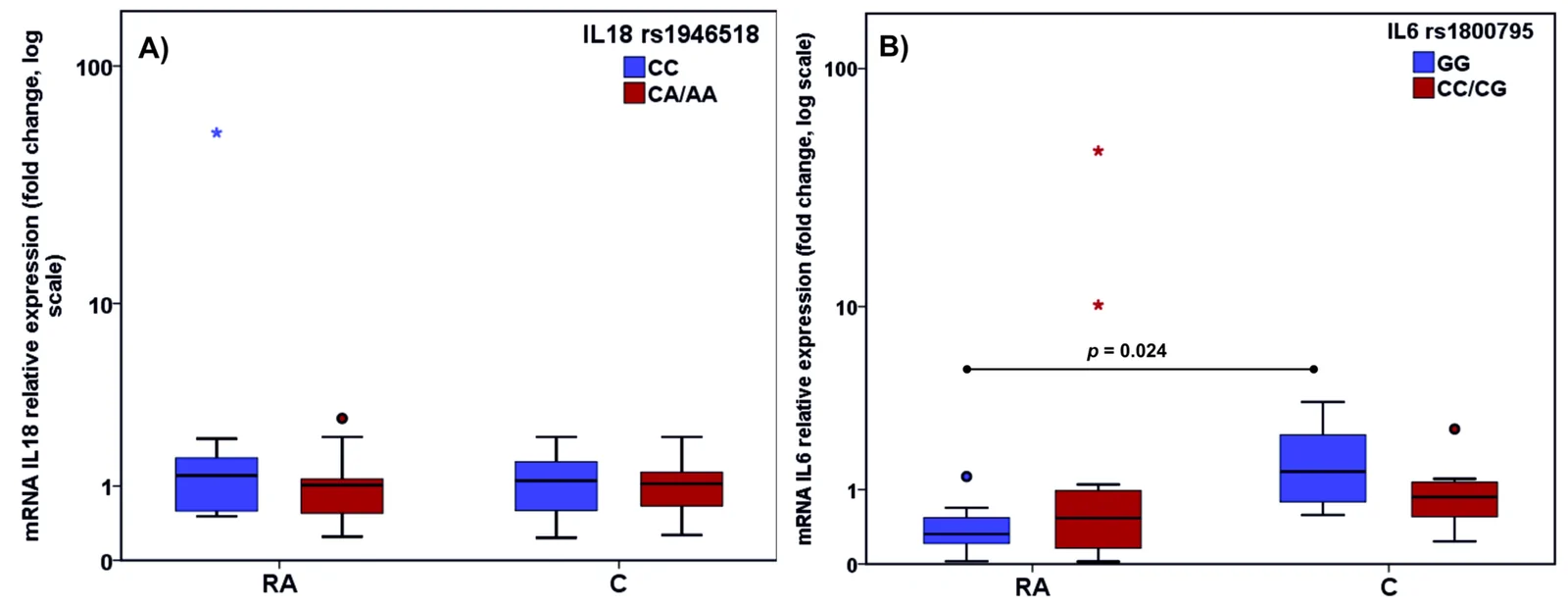
Relative mRNA expression levels of *IL18* (**A**) and *IL6* (**B**) in female patients with rheumatoid arthritis (RA) and healthy controls (C), stratified according to the *IL18* rs1946518 and *IL6* rs1800795 genotypes. Data are presented as the median with the lower (Q1) and upper (Q3) quartiles. Outliers are indicated by circles, and extreme values are indicated by asterisks. The *y*-axis is displayed on a logarithmic scale.

The relative mRNA expression levels of IL6 and IL18 were examined in a limited group of female RA patients (n = 33) and controls (n = 23). Data are presented in Figure 1. The mRNA expression of IL18, both when comparing RA patients to controls overall (Figure 1A) and when stratified by the *IL18* rs1946518 genotypes (CC vs. CA/AA) (Figure 2A), showed no notable differences between the groups. In contrast, analysis of *IL6* mRNA expression (Figure 1B) revealed a statistically significant difference between the case and control groups (*p* = 0.037). IL6 expression was lower in the RA cohort than in control subjects. When examining the association with the IL6 rs1800795 polymorphism, a significant difference was found. Specifically, among control subjects, individuals carrying the GG genotype exhibited significantly higher IL6 expression than RA patients with the same genotype (*p* = 0.024). Higher IL6 mRNA expression was observed in controls with the GG genotype compared to those with the CC/CG genotypes (Figure 2B), although this difference did not reach statistical significance.

## 3. Discussion

In the present study, we examined how the *IL6* (rs1800795) and *IL18* (rs1946518) promoter polymorphisms affect disease susceptibility, circulating cytokine levels, and gene expression in patients with RA from the Bulgarian population. The main findings indicate that although serum IL-6 and IL-18 levels were significantly elevated in RA patients compared to healthy controls, rs1800795 demonstrated only borderline associations with disease risk and autoantibody status, whereas rs1946518 was not linked to disease susceptibility, cytokine levels, or major clinical characteristics of RA. Although the present study included 159 patients with rheumatoid arthritis and 188 healthy controls, sensitivity analysis indicated that the study had adequate statistical power (80%, α = 0.05) to detect moderate or larger genetic effects. The observed effect sizes for the investigated polymorphisms were small (Cohen’s w = 0.073 for IL18 rs1946518 and w = 0.091 for IL6 rs1800795), suggesting that the true genetic effects, if present, are likely modest. Therefore, the absence of statistically significant associations should be interpreted with caution, as the study was not powered to reliably detect very small genetic effects. Future larger multicenter studies and meta-analyses are warranted to further clarify the contribution of these promoter polymorphisms to rheumatoid arthritis susceptibility.

The observed elevation in circulating IL-18 levels in RA patients is consistent with previous reports demonstrating the important role of this cytokine in the pathogenesis of autoimmune inflammatory diseases. IL-18 is a member of the IL-1 cytokine family and is produced following activation of inflammasome complexes. Its maturation is tightly linked to inflammasome activation, particularly through the NLRP3–caspase-1 pathway. IL-18 maturation depends on caspase-1 activation during inflammasome signaling, a process closely associated with inflammatory cell death known as pyroptosis. Previous studies have reported upregulation of NLRP3, caspase-1 and cleaved GSDMD in RA synovial macrophages [14] as well as significantly higher relative mRNA expression levels of NLRP3, *NLRC4*, *AIM2*, caspase-1, and IL-1beta in PBMC of RA patients [15]. In addition, Jiang et al. reported no significant difference in IL-18 mRNA expression (*p* = 0.782) but observed markedly higher plasma IL-18 levels (*p* = 0.001) in RA cases compared with controls. These findings are consistent with those of the present study and suggest that inflammasome activation and pyroptotic pathways mainly contribute to local synovial inflammation. Elevated serum IL-18 levels observed here may reflect activation of local inflammasome-driven inflammatory pathways.

An important finding of this study is the sex difference in serum IL-18 levels, with healthy men exhibiting higher IL-18 levels than healthy women, suggesting that sex hormones or sex-specific regulation influences IL-18 biology. This observation aligns with some data in the literature showing sex differences in IL-18 and NLRP3 inflammasome activity, as sex hormones modulate the priming and activation phases of the NLRP3 pathway [16]. In many physiological settings, estrogen suppresses activation of the NLRP3 inflammasome and production of pro-inflammatory cytokines IL-1β and IL-18 [17], whereas androgens promote and sustain NLRP3 inflammasome pathways in some immune cells [18]. When examining the *IL18* promoter polymorphism rs1946518 (−607 C > A), individuals carrying the C allele in the genotype tended to have higher IL-18 levels than those with the AA genotype in the overall healthy control cohort. The −607 A allele has been linked to altered transcription factor binding, lower promoter activity and lower serum IL-18 levels [19]. Conversely, the −607 C allele has been associated with higher circulating IL-18 levels in relation to different diseases [20,21]. However, in our analysis confined to healthy women, this tendency for the C allele to be associated with increased IL-18 was significantly weaker. These findings suggest that the relationship between rs1946518 and IL-18 production may be influenced by sex, with a stronger association observed in men than in women. In women, estrogen or other regulatory pathways might normalize IL-18 production across genotypes, diminishing the detectable difference between CA and AA. Overall, our results support the involvement of both sex and genetics in regulating IL-18 levels in healthy individuals, highlighting the importance of considering sex as a key stratification factor in IL-18-related studies. The sex differences in IL-18 levels and the impact of *IL18* −607 C/A on IL-18 production across genotypes in our healthy cohort were not evident in RA patients. These findings suggest that the pathogenic mechanisms of RA may differ between men and women, and that deregulation of serum IL-18 levels could be a vulnerable node, particularly impactful in female RA patients. On the other hand, we can assume that chronic inflammation in RA likely influences the fine genetic regulation of IL-18 expression.

While IL-18 is undoubtedly associated with RA inflammation, our data on the rs1946518 SNP and disease susceptibility did not identify an impact of the *IL18* −607 C/A SNP on disease susceptibility. Allelic and genotypic frequencies of the *IL18* −607 C/A SNP did not differ between RA patients and healthy controls in our population, and there was no association with disease characteristics. Many studies have focused on this polymorphism, but findings on its role in disease susceptibility and development are inconsistent and controversial. Several studies have found no significant association between rs1946518 and susceptibility to RA [22,23,24,25,26]. In contrast, some studies have shown that the A allele, or AA/AC genotypes, may confer protection against RA in certain populations, particularly in Asian cohorts [27,28,29,30]. Conversely, the C allele and the CC/AC genotypes are risk factors and may be linked to greater erosive disease and higher inflammatory markers in RA patients [30,31]. However, other studies report no association between this polymorphism and factors such as sex, age at onset, or RF status [27]. The inconsistency in findings suggests that the impact of rs1946518 on RA may be dependent on the population studied, study sample size, and the genetic background. Several meta-analyses have been conducted to clarify whether this SNP influences the genetic risk of developing RA: some meta-analyses found no significant association in the overall population [6,32], while others suggest the protective effect of the A allele or the AA genotype of rs1946518 in the Chinese population but not in Caucasian subgroups, indicating potential ethnic differences [29,33]. Recently, an umbrella review of meta-analyses was conducted, with data re-analysed [13]. The results suggest that the *IL18* −607 C/A polymorphism is associated with a reduced risk of developing RA across the overall population in allele, dominant, homozygote and heterozygote models. However, the authors identify several limitations of the included meta-analyses and emphasise the need for updated, larger-scale studies to determine the precise role of this polymorphism in RA.

The *IL6* −174 G/C (rs1800795) has been intensively studied and was associated with susceptibility to several diseases, including RA. Despite extensive research, the evidence regarding the association between the −174 G/C polymorphism and the risk or severity of rheumatoid arthritis remains inconsistent across different ethnic groups. In Caucasian populations, meta-analyses generally report no significant association between rs1800795 and RA susceptibility [34,35]. In contrast, this polymorphism appears to be a significant risk factor for RA in Asian populations, where the C allele is associated with increased risk [36]. Similar findings were recently reported by Hao et al. in a meta-analysis of 21 studies comprising 9772 participants, including 4679 patients with RA and 5093 healthy controls. Stratified analyses based on ethnicity (Asian, European, Caucasian, Southeast Asian, and mixed populations) revealed a significant association between the *IL6* rs1800795 and RA susceptibility only among Asian populations, specifically for the heterozygous CG genotype [37]. However, in Caucasian cohorts, the rs1800795 variant has a more prominent role in disease severity and clinicopathological features of RA rather than initial susceptibility [3]. Our results indicate that carrying the G allele and the GG + CG genotypes may predispose to RA, whereas the homozygous CC genotype may be protective against RA. RA patients carrying the *IL6* −174 G allele (CG + GG) were more likely to be RF-positive than those with the homozygous CC genotype (75.0% vs. 53.3%, OR = 1.4, 95% CI = 0.86–2.28, *p* = 0.072).

Among healthy controls, the rs1800795 polymorphism significantly influenced *IL6* mRNA expression, with individuals carrying the GG genotype showing higher expression levels (*p* = 0.024). These results are in agreement with previous studies on the in vivo and in vitro functional significance of the −174 G/C variant in *IL6.* The rs1800795 polymorphism is located in the proximal promoter region of the *IL6* gene and is thought to influence the binding of the glucocorticoid receptor, thereby repressing transcriptional activation. The rs1800795 C allele has been shown to exhibit lower IL-6 transcriptional activity and is associated with reduced expression levels compared to the G allele [37,38,39].

In patients with RA, no clear association was observed between the *IL6* −174 G/C polymorphism and either *IL6* mRNA expression or circulating IL-6 levels. One possible explanation is that the effect of this genetic variant is masked by the highly complex inflammatory environment characteristic of RA. Furthermore, IL-6 plays a pleiotropic role in the immunopathogenesis of RA. In combination with TGF-β, IL-6 stimulates naïve T-cells to differentiate into pro-inflammatory Th17 cells, a subset implicated in the initiation and progression of chronic inflammation and autoimmune disease. This process is mediated primarily through activation of the JAK/STAT3 signalling pathway. Activated STAT3 induces the expression of the lineage-defining transcription factors RORγt and RORα, which are essential for Th17-cell differentiation and the production of IL-17 [40]. IL-6 contributes not only to the initiation of pathogenic immune responses but also to the maintenance of chronic inflammation and joint destruction in RA and its comorbidities [41,42,43]. Therefore, disease-related activation of IL-6-dependent signalling networks may outweigh the relatively modest effects of the −174 G/C polymorphism on IL-6 expression, rendering genotype-dependent differences difficult to detect in patients with established RA.

Regarding *IL6* quantity, our results showed lower IL-6 mRNA in RA peripheral blood despite higher serum levels, which seems paradoxical but is not surprising. Circulating IL-6 could derive from many cellular sources, including infiltrating immune cells within inflamed joints, rather than transcriptional activity in peripheral blood cells. In addition, IL-6 expression can be regulated at both a transcriptional and a post-transcriptional level, including mRNA stability, translational efficiency, protein secretion, and cytokine clearance. In addition, the observed discrepancy may also have been influenced by antirheumatic treatment, as csDMARDs, bDMARDs, and glucocorticoids are known to modulate cytokine production and gene expression. Furthermore, differences in the cellular composition of peripheral blood mononuclear cells (PBMCs) between patients and controls may also have contributed to the reduced *IL6* mRNA expression observed in peripheral blood. Several studies have investigated the regulatory role of miRNAs on the production of cytokines, and disturbances in the balance between Th17 and Treg cells in RA [44,45]. Consequently, elevated circulating IL-6 may persist even when *IL6* transcription in peripheral blood cells is reduced.

Our study is the first to examine the association of rs1946518 (*IL18* −607 C/A) and rs1800795 (*IL6* –174 G/C) with RA susceptibility in a Bulgarian cohort, integrating genetic data with protein levels, mRNA expression, and clinical data. However, some limitations of our study should be acknowledged. Although we considered several confounding factors, the cross-sectional design prevents us from ruling out the potential impact of medications (csDMARDs/bDMARDs) on cytokine levels. Additionally, there was a noticeable imbalance in the male-to-female ratio between the rheumatoid arthritis group and the healthy controls. The distribution largely reflects the well-known female predominance in rheumatoid arthritis. This sex imbalance between the rheumatoid arthritis and healthy control groups is a potential limitation, particularly given the observed sex-related differences in IL-18 levels. Although sex-adjusted analyses did not materially alter the results, the potential influence of sex on cytokine expression should be considered when interpreting the findings. Future research involving larger, more balanced cohorts with increased male participation is necessary to confirm these findings. Additionally, the sample size for the mRNA analysis was limited which restricted the ability to draw robust conclusions and warrants further validation in larger cohorts. Also, we focused on two key genetic variants in the promoters of the target genes. While these variants have well-established functional effects, other variants within the same genes or in related genes might also affect their expression at both the mRNA and protein levels.

## 4. Materials and Methods

### 4.1. Study Subjects

In this cross-sectional study, we enrolled consecutive patients diagnosed with rheumatoid arthritis who visited the Rheumatology Clinic of University Hospital “St. Ivan Rilski” in Sofia between October 2016 and October 2022. The inclusion criteria for patients with RA were individuals 18 years or older who satisfied the 2010 RA classification criteria established by the American College of Rheumatology (ACR) and the European League Against Rheumatism (EULAR) [46]. Clinical assessment included collecting demographic data and obtaining information regarding disease activity, autoantibody status, and therapy for RA. Anti-CCP antibodies were detected using an ELISA (REF EA1505–9601G; EUROINMUN, Lubeck, Germany). Individual disease activity was quantified using the Disease Activity Score for 28 joints, based on C-reactive protein (DAS28-CRP). The exclusion criteria included: no history of other inflammatory rheumatological or autoimmune disorders, no malignancy, body mass index (BMI) of 29.9 or higher, no significant unstable or uncontrolled acute or chronic disease, and no current active infection. Comparisons were made with healthy controls (HCs) who were unrelated to each other and selected from university and hospital staff. The exclusion criteria were the same as mentioned previously. The study was approved by the local Ethics Committee of the University Hospital “St. Ivan Rilski” in Sofia, Bulgaria, with decision number 6, dated 29 November 2016, and the Ethics Committee of the Medical Faculty, Trakia University (protocol 16/19.03.2021). All subjects signed informed consent in accordance with the ethical guidelines of the Helsinki Declaration.

### 4.2. Blood Samples and DNA Extraction

Blood samples from RA patients and controls were collected in Gel/clot activator vacutainer tubes. The blood samples were allowed to clot for 30 min at room temperature before being centrifuged. Serum samples were collected and frozen at −70 °C for enzyme-linked immunosorbent assay (ELISA) analysis. Peripheral blood was collected in vacutainer tubes containing ethylenediaminetetraacetic acid (EDTA) as an anticoagulant for DNA and RNA isolation.

Genomic DNA was extracted using a genomic blood DNA purification kit (NucleoSpin Blood L, Macherey-Nagel, Duren, Germany) and stored at −80 °C until use. The NanoVuetm Spectrophotometer 9 (GE Healthcare, Buckinghamshire, UK) was used to measure the concentration of the resulting DNA spectrophotometrically at 260 nm. The purity of DNA samples was assessed by comparing the absorbance at 260 nm to that at 280 nm.

### 4.3. Genotyping Analysis

Genotyping for the −607 C/A polymorphism in the IL18 gene (rs1946518) was performed by an amplification refractory mutation system (ARMS)–PCR assay. In this method, a primer specific to the C allele was used, 5′GTTGCAGAAAGTGTAAAAATTATTAC3′, a forward primer specific for the A allele, 5′GTTGCAGAAAGTGTAAAAATTATTATTAA3′, and reverse generic primer 5′TAACCTCATTCAGGACTTCC3′. Reactions were carried out in 0.2 mL microtubes, and the final volume was 20 μL including 0.2 mM dNTP, 0.3 μM common reverse primer and forward primer, 2.5 mM MgCl_2_, 1U TaqDNA polymerase, 10 μL 10 × PCR-buffer and 0.2 μg genomic DNA. PCR was carried out using a thermocycler GeneAmp PCR System 9700 (Applied Biosystems, Foster City, CA, USA) set to the following thermal conditions: a denaturation step for 5 min at 95 °C followed by 35 cycles of denaturation for 30 s at 94 °C, annealing for 30 s at 56 °C and extension for 45 s at 72 °C and finally an extension step for 5 min at 72 °C. Amplification products were analyzed using electrophoresis in 2% agarose gels, detected via staining with ethidium bromide and reported on the Herolab gel documentation system.

To verify the genotyping analysis, we used a second analysis: polymerase chain reaction-restriction fragment length polymorphism (PCR-RFLP) assay. Amplification of the 301 bp fragment was performed with the 5′TAACCTCATTCAGGACTTCC3′ and 5′CTTTGCTATCATTCCAGGAA3′ primers at the following cycling parameters: initial incubation step of 5 min at 95 °C; 35 cycles of 30 s at 94 °C, 30 s at 58 °C, and 45 s at 72 °C; and a final extension step of 5 min at 72 °C to complete the reaction. The amplified products (15 μL) were digested using 10 U restriction enzyme Tru1I (Msel; Tru9I) per reaction for 12 h at 65 °C. The products were analyzed using electrophoresis in 3% agarose gels on the Herolab gel documentation system and its analysis software E.A.S.Y. Win 32 (Herolab GmbH Laborgeräte, Wiesloch, Germany). The −607 C allele yields three fragments at 199 bp, 73 bp and 29 bp. The −607 A allele yields four fragments at 73 bp, 101 bp, 98 bp, and 29 bp.

A heterozygous control template was used to ensure accuracy in each PCR run. For quality control, 10% of randomly selected samples including both cases and controls were reanalyzed without any discrepancies.

Polymorphism at position −174 C/G in the promoter region of the IL-6 gene (rs1800795) was also genotyped by ARMS-PCR. Primers were one common 5′GAGCTTCTCTTTCGTTCC3′ and two allele-specific primers, one for the C allele, 5′CCCTAGTTGTGTCTTGCC3′, and the second for the G allele, 5′CCCTAGTTGTGTCTTGCG3′. The reaction was performed in a single tube with a final reaction volume of 20 μL including 0.2 mM dNTP, 0.4 μM common reverse primer and forward primer, 1U TaqDNA polymerase, 10 μL 10 × PCR-buffer and 0.2 μL genomic DNA. PCR was carried out using a thermocycler set to the following thermal conditions: a denaturation step for 5 min at 95 °C followed by 30 cycles of denaturation for 30 s at 94 °C, annealing for 60 s at 50 °C and extension for 60 s at 72 °C and finally an extension step for 7 min at 72 °C. Amplification products were analyzed by electrophoresis on 2% agarose gels, stained with ethidium bromide, and documented on the Herolab gel documentation system.

### 4.4. Gene Expression Analysis

Peripheral venous blood samples served as the source material for gene expression analysis. Total RNA was isolated using the Thermo Scientific GeneJET RNA Purification Kit (Thermo Fisher Scientific Inc., Vilnius, Lithuania) following the manufacturer’s instructions. The yield and purity of the extracted RNA were evaluated spectrophotometrically. Subsequently, 1 μg of RNA was reverse-transcribed into complementary DNA (cDNA) with the RevertAid First Strand cDNA Synthesis Kit (Thermo Fisher Scientific Inc.) using random hexamer primers.

Quantitative real-time PCR (qPCR) was performed using TaqMan inventoried assays for the target genes *IL6* and *IL18* and the reference genes 18S rRNA and GAPDH. The assay IDs were as follows: for *IL18*—Hs01038788_m1; *IL6*—Hs00985639_m1; *GAPDH:* Hs02758991_g1; and 18S rRNA: Hs999901_s1. Thermocycling conditions included an initial step at 95 °C for 10 min, followed by 40 cycles of denaturation at 95 °C for 15 s and annealing/extension at 60 °C for 1 min. A 7500 Real-Time PCR System and 7500 Software v 2.3 (Applied Biosystems, Foster City, CA, USA) were used.

Gene expression levels were normalized to the reference genes, and relative quantification was calculated using the ΔCt method. Results are reported as the fold changes relative to the calibrator group.

### 4.5. Quantification of Serum Cytokine Concentrations

Serum levels of IL-6 and IL-18 were quantified using commercially available ELISA kits, following the manufacturer’s instructions. The IL-6 kits were purchased from Elabscience (Houston, TX, USA) and Human IL-18 ELISA kits were purchased from eBioscience (Vienna, Austria). A standard curve (5-parametric model) constructed using the kits’ standards was used to determine the concentration of cytokines expressed in picograms per millilitre (pg/mL). Serum samples from patients and controls were analysed in duplicate within the same kit’s batch. The lower limit of detection (LLOD) was 0.94 pg/mL for IL-6 and 9 pg/mL for IL-18. Values below the LLOD were present only for IL-6, and OD values equal to zero were set to 0.1 pg/mL.

### 4.6. Data Analysis

All data for this study were analyzed using RStudio Verison 2026.01.2 (R programming language) and SPSS version 28.0 for Windows (SPSS Inc., Chicago, IL, USA). The normality of the distributions was evaluated using the Kolmogorov–Smirnov and Shapiro–Wilk tests. The goodness of fit to the Hardy–Weinberg equilibrium (HWE), calculating the expected frequencies of each genotype and comparing them with the observed values for patients and healthy controls, was performed using a chi-squared test. Six inheritance models, including an allelic model with the wild-type (common) allele as the reference, as well as co-dominant, dominant, recessive, over-dominant, and log-additive genetic models, were used to evaluate the impact of individual alleles and genotypes on the disease prevalence. Differences in study cytokine concentrations between RA patients and healthy controls and different genotypes were evaluated using Mann–Whitney U tests. Disease characteristics of RA in relation to *IL18* −607 C/A (rs1946518) and *IL6* −174 C/G (rs1800795) SNPs were assessed using the Mann–Whitney U test, chi-squared test, and binary logistic regression analysis as appropriate. General linear models were used to assess the relationship between the natural logarithm of circulating IL-6 and IL-18 with the levels of major study variables and studied SNPs. A two-tailed *p* value of <0.05 was considered significant.

## 5. Conclusions

Although our results revealed only modest or borderline associations between rs1946518 and rs1800795 and certain clinical characteristics of RA, neither variant was significantly associated with RA susceptibility in our cohort. Furthermore, the functional effects of these polymorphisms on cytokine expression appeared to be attenuated in the context of the complex inflammatory environment characteristic of RA, where disease-related regulatory mechanisms may outweigh the contributions of individual genetic variants. Nevertheless, our findings support the pivotal role of IL-6 and IL-18 in RA pathogenesis and highlight the importance of considering sex as a key biological variable in studies of autoimmunity and immune-mediated diseases.

## Figures and Tables

**Table 1:** Demographic characteristics and clinical data of RA patients.

	RA	Controls	*p*
n	159	188	
Age (years)	55.4 ± 11.8	52.8 ± 12.4	0.051
Sex, n (%)			
males	11 (6.9%)	66 (35.1%)	<0.001
females	148 (93.1%)	122 (64.9%)	
Disease duration (years)	8.9 ± 8.6		
RF-IgM-positive cases (n, %)	116 (73%)		
Anti-CCP-positive cases (n, %)	97 (61.0%)		
CRP (mg/L)	21.5 ± 40.2		
DAS28-CRP	4.75 ± 1.3		
low activity/remission	18 (11.3%)		
moderate activity	94 (59.1%)		
high activity	47 (29.6%)		
Therapy			
Symptomatic (%)	53 (33.3%)		
csDMARDs (%)	83 (52.2%)		
bDMARDs (%)	23 (14.5%)		

Data are presented as mean ± SD. CRP, C reactive protein; DAS28-CRP, Disease Activity Score 28 calculated using CRP; DMARDs, disease-modifying anti-rheumatic drugs, csDMARDs, conventional synthetic; RA, rheumatoid arthritis; RF, rheumatoid factor; SD, standard deviation.

**Table 2:** Allele and genotype frequencies of *IL18* −607 C/A and IL6-174 C/G SNPs in RA patients and healthy controls.

Locus		RA n = 159 (%)	Controls n = 188 (%)	OR (95% CI) *	*p* Value
***IL18*** −**607 C/A (rs1946518)**					
	Genotype				
Co-dominant model	CC	58 (36.5)	82 (43.6)	ref.	
	CA	85 (53.5)	90 (47.9)	1.20 (0.75–1.94)	0.72
	AA	16 (10.0)	16 (8.5)	1.23 (0.55–2.73)	
Dominant model	CC	58 (36.5)	82 (43.6)	ref.	
	CA + AA	101 (63.5)	106 (56.4)	1.21 (0.76–1.91)	0.42
Recessive model	CC + CA	143 (89.9)	172 (91.5)	ref.	
	AA	16 (10.1)	16 (8.5)	1.11 (0.52–2.36)	0.79
Over-dominant model	CC + AA	74 (46.5)	98 (52.1)	ref.	
	CA	85 (53.5)	90 (47.9)	1.16 (0.74–1.81)	0.53
Log-additive model				1.14 (0.80–1.63)	0.46
HWE		χ^2^ = 3.54 *p* = 0.06	χ^2^ = 1.59 *p* = 0.21		
***IL6* −174 C/G (rs1800795)**					
	Genotype				
Co-dominant model	GG	69 (43.4)	79 (42.0)	ref.	
	CG	75 (47.2)	80 (42.6)	1.00 (0.62–1.62)	0.23
	CC	15 (9.4)	29 (15.4)	0.55 (0.26–1.1506)	
Dominant model	GG	69 (43.4)	79 (42.0)	ref.	
	CG + CC	90 (56.6)	109 (58.0)	0.88 (0.56–1.39)	0.58
Recessive model	GG + CG	144 (90.6)	159 (84.6)	ref.	
	CC	15 (9.4)	29 (15.4)	0.55 (0.27–1.10)	0.087
Over-dominant model	CC + GG	84 (52.8)	108 (57.5)	ref.	
	CG	75 (47.2)	80 (42.5)	1.14 (0.73–1.8)	0.56
Log-additive model				0.81 (0.58–1.13)	0.22
HWE		χ^2^ = 0.72 *p* = 0.48	χ^2^ = 1.29 *p* = 0.26		

HWE, Hardy–Weinberg equilibrium; RA, rheumatoid arthritis; OR, odds ratio; CI, confidence interval. * adjusted by sex and age.

**Table 3:** Disease characteristics of RA in relation to *IL18* −607 C/A (rs1946518) and *IL6* −174 C/G (rs1800795) SNPs. Data are presented as mean ± SD or percentage of positivity. Comparisons between groups were made by the Mann–Whitney U test or chi-squared test, as appropriate.

	rs1946518			rs1800795		
Parameter	CC	CA + AA	*p*-Value	GG + CG	CC	*p*-Value
Age (years)	55.0 ± 11.3	55.63 ± 12.1	0.635	55.4 ± 11.7	55.5 ± 13.0	0.981
Disease onset						
young-onset	48.3%	47.5%	0.927	28.5%	13.3%	0.209
late-onset	51.7%	52.5%		71.5%	86.7%	
Disease duration (years)	8.5 ± 9.4	9.2 ± 8.2	0.241	9.1 ± 8.8	7.4 ± 6.9	0.633
CRP	21.2 ± 34.4	21.7 ± 43.4	0.47	22.3 ± 41.84	13.6 ± 14.9	0.566
DAS28-CRP	4.7 ± 1.1	4.6 ± 1.3	0.689	4.7 ± 1.2	4.5 ± 1.4	0.449
RF status positive	70.7%	74.3%	0.626	75.0% G allele in genotype	53.3% OR = 1.4 (95% CI)	0.072 0.86–2.28
CCP status positive	65.5%	58.4%	0.377	61.1%	60.0%	0.933

CRP, C-reactive protein; DAS28-CRP, Disease Activity Score 28 calculated using C-reactive protein level; CCP, cyclic citrullinated peptide antibody; RA, rheumatoid arthritis; RF, rheumatoid factor; SD, standard deviation.

**Table 4:** Serum cytokine levels in RA patients and healthy controls.

Cytokine	RA	Controls	*p* *
IL-18 pg/mL	222.9 ± 265.6 153.4 (132.4)	128.9 ± 76.7 106.2 (118.9)	<0.001
male	220.3 ± 143.4 210.5 (246.1)	161.7 ± 75.4 168.6 (132.5)	0.204
female	223.2 ± 273.5 152.0 (116.6)	96.7 ± 63.5 76.4 (83.6)	<0.001
*p*	0.573 *	<0.001 *	
IL-6 pg/mL	23.6 ± 38.9 8.1 (23.2)	1.8 ± 2.4 1.2 (2.3)	<0.001
male	51.2 ± 70.2 11.1 (118.6)	2.6 ± 3.3 1.4 (2.8)	0.004
female	20.1 ± 32.8 8.1 (20.6)	1.3 ± 1.2 0.9 (1.9)	<0.001
*p*	0.443 *	0.107 *	

* Mann–Whitney U test, RA, rheumatoid arthritis; data are presented as mean ± SD and median (IQR).

**Table 5:** Serum levels of IL-18 and IL-6 in RA patients and healthy controls in relation to *IL18* −607 C/A and *IL6* −174 C/G SNPs, respectively.

	RA	Controls	*p* Value *
	IL-18 (pg/mL)	IL-18 (pg/mL)	
rs1946518 genotypes			
CC	235.8 ± 302.0	132.1 ± 76.4	0.014
	158 (129.8)	114.9 (112.6)	
CA	207.4 ± 257.5	143.4 ± 78.1	0.166
	145 (117.8)	135.0 (131.2)	
AA	280.3 ± 235.3	88.0 ± 56.4	0.009
	172.7 (369.2)	73.4 (55.8)	
*p* value within the group **	0.302	0.183	
	IL-6 (pg/mL)	IL-6 (pg/mL)	
rs1800795 genotypes			
GG	23.3 ± 40.4	2.0 ± 3.2	<0.001
	6.2 (15.9)	1.3 (2.3)	
CG	24.5 ± 40.5	1.9 ± 2.2	<0.001
	9.6 (24.6)	1.3 (2.0)	
CC	23.4 ± 35.1	0.38 ± 0.3	0.012
	8.9 (36.9)	0.38 (0.6)	
*p* value within the group **	0.572	0.148	

* Mann–Whitney U test, ** Kruskal–Wallis test, RA, rheumatoid arthritis; results are presented as mean ± SD and median (IQR).

**Table 6:** Serum levels of IL-18 and IL-6 in female RA patients and healthy women in relation to *IL18* −607 C/A and *IL6* −174 C/G SNPs, respectively.

	RA	Controls	*p* Value *
	IL-18 (pg/mL)	IL-18 (pg/mL)	
rs1946518 genotypes			
CC	245.5 ± 310.3	107.9 ± 65.6	0.002
	158.0 (160.8)	86.0 (95.5)	
CA	205.1 ± 265.4	102.2 ± 67.4	0.002
	143.2 (114.34)	85.2 (97.0)	
AA	266.5 ± 237.8	87.9 ± 61.8	0.02
	171.2 (302.0)	69.0 (83.8)	
*p* value within the group **	0.281	0.813	
	IL-6 (pg/mL)	IL-6 (pg/mL)	
rs1800795 genotypes			
GG	21.2 ± 35.7	1.0 ± 1.4	<0.001
	6.2 (15.9)	0.59 (1.4)	
CG	20.2 ± 31.9	1.3 ± 1.0	<0.001
	8.5 (24.5)	1.2 (1.5)	
CC	12.8 ± 16.1	0.47 ± 0.4	<0.001
	6.9 (27.9)	0.5 (0.3)	
*p* value within the group **	0.560	0.219	

* Mann-Whitney U test, ** Kruskal–Wallis test, RA, rheumatoid arthritis; results are presented as mean ± SD and median (IQR).

## Data Availability

The data presented in this study are available upon request from the corresponding author. The data are not publicly available due to local regulation and hospital restrictions.

## Supplementary Materials

The following supporting information can be downloaded at: https://www.mdpi.com/article/10.3390/ijms27167132/s1.
