# Supplementary material for: Impact of IL6 and IL18 Promoter Polymorphisms on Circulating Cytokine Levels, Gene Expression, and Susceptibility to Rheumatoid Arthritis
Source: Int J Mol Sci. 2026 Aug 9;27(16):7132. doi: 10.3390/ijms27167132 (PMC13513842; doi:10.3390/ijms27167132)
Supplement: Supplementary file 1 [file ijms-27-07132-s001.zip › ijms-4438895-supplementary.pdf]

Supplementary Table S1. Effect of the combined *IL18 −607 C/A* and *IL-6 −174 C/G* genotypes on the RA susceptibility.

IL18 −607 C/A	*IL6 −174 C/G*	*RA* n=159 (%)	*Controls* n=188 (%)	*OR (95 % CI)*	*p value*
CC	GG/CG	53 (33.3)	70 (38.3)	ref	
CC	CC	5 (3.1)	15 (8.2)	0.44 (0.15-1.29)	0.13
CA/AA	GG/CG	91 (57.2)	86 (47.0)	1.398 (0.88 -2.22)	0.156
CA/AA	CC	10 (6.3)	12 (6.6)	1.1 (0.44-2.74)	0.836

RA: rheumatoid arthritis; OR: odds ratios; CI: confidence intervals

Supplementary Table S2. Multivariate relationship between study gene polymorphism of *IL18-607* C/A (rs1946518) sex, and age with IL-18 serum levels in healthy controls established by a general linear model of log-transformed levels of IL-18 with bootstrap resampling (1000 samples).

**Independent Variable**	**Dependent Variable: ln IL-18 pg/mL**			
	**Parameter Estimate (SE)**	***p*-Value**	**Partial Eta^2**	**Power**
IL18-607 C/A				
CC	0.188 (0.27)	*0.28*	0.004	0.106
CA	0.267 (0.27)	*0.125*	0.009	0.166
AA	Reference			
Sex				
male	0.487	*0.000*	0.122	0.972
female	Reference			
Age (years)	-0.003 (0.005)	*0.599*	0.003	0.082

Supplementary Table S3. Multivariate relationship between study gene polymorphism of *IL6-174*C/G (rs1800795) sex, and age with IL-6 serum levels in healthy controls, established by a general linear model of log-transformed levels of IL-6 with bootstrap resampling (1000 samples).

**Independent Variable**	**Dependent Variable: ln IL-6 pg/mL**			
	**Parameter Estimate (SE)**	***p*-Value**	**Partial Eta^2**	**Power**
IL6-174 C/g				
GG	0.927 (0.701)	0.191	0.025	0.256
CG	1.152 (0.682)	0.096	0.040	0.384
CC (n=5)	Reference			
Sex				
male	0.503 (0.346)	0.150	0.030	0.301
female	Reference			
Age (years)	0.30 (0.016)	*0.073*	0.047	0.4536
